# Strain-induced yellow to blue emission tailoring of axial InGaN/GaN quantum wells in GaN nanorods synthesized by nanoimprint lithography

**DOI:** 10.1038/s41598-021-86139-9

**Published:** 2021-03-24

**Authors:** Geoffrey Avit, Yoann Robin, Yaqiang Liao, Hu Nan, Markus Pristovsek, Hiroshi Amano

**Affiliations:** 1 IMaSS, Nagoya University, Furo-cho, Chikusa-ku, Nagoya, 464-8601 Japan; 2 Present Address: CNRS, UMR6602, Institut Pascal, 4 Avenue blaise Pascal, 63178 Aubière, France

**Keywords:** Nanowires, Nanoscale materials

## Abstract

GaN nanorods (NRds) with axial InGaN/GaN MQWs insertions are synthesized by an original cost-effective and large-scale nanoimprint-lithography process from an InGaN/GaN MQWs layer grown on c-sapphire substrates. By design, such NRds exhibit a single emission due to the c-axis MQWs. A systematic study of the emission of the NRds by time-resolved luminescence (TR-PL) and power dependence PL shows a diameter-controlled luminescence without significant degradation of the recombination rate thanks to the diameter-controlled strain tuning and QSCE. A blueshift up to 0.26 eV from 2.28 to 2.54 eV (543 nm to 488 nm) is observed for 3.2 nm thick InGaN/GaN QWs with an In composition of 19% when the NRds radius is reduced from 650 to 80 nm. The results are consistent with a 1-D based strain relaxation model. By combining state of the art knowledge of c-axis growth and the strong strain relieving capability of NRds, this process enables multiple and independent single-color emission from a single uniform InGaN/GaN MQWs layer in a single patterning step, then solving color mixing issue in InGaN based nanorods LED devices.

## Introduction

InGaN based semiconductors have a direct bandgap that can be tuned across the entire visible spectrum, from 0.7 eV for InN to 3.4 eV for GaN. Efficient blue and green emitting lasers and light emitting diodes (LEDs) have been achieved for many years^1–3^. However, InGaN must be grown at relatively low temperature which results in poor crystalline quality when the InGaN/GaN quantum wells (QWs) reach high In composition (> 20%)^4–7^. This hinders the development of efficient red emitting diodes. Furthermore, the compressive strain due to the lattice mismatch between InGaN and GaN induces a large piezoelectric field, which increases with the In composition. This reduces the radiative recombination rate due to spatially separating electron and hole wavefunctions, and induces a shift of the emission towards longer wavelength—the quantum confined Stark effect (QCSE). QSCE together with non-radiative recombinations at defects is also assumed as main causes for the reduced internal quantum efficiency (IQE)^4^ in InGaN based LEDs towards longer emission wavelengths. Interestingly, despite the strong QCSE, the luminescence of InGaN heterostructures on (0001) planes shows the highest IQE compared to m-axis or semipolar orientations^3,8,9^. Recently, incorporating AlGaN layers into the QW barriers increased red emitting InGaN based LEDs more likely^5,10,11^. Hwang et al.^12^ demonstrated strong red luminescence with a peak wavelength at 629 nm and FWHM of 60 nm using an InGaN/AlGaN QW structure. Delta growth of AlN and InN has also shown some promising results^13^.

Another approach is the use of nanostructures and nanorods (NRds), which are promising for the integration of high efficiency LEDs devices^14–19^, for instance into micro-displays. NRds relieve the strain of vertical InGaN QWs at their sidewalls; and core–shell structures offer large active surface. Nevertheless, standard core/shell structures obtained by selective area growth (SAG) on masked substrates often have three or more type of facets (m-planes, c-plane and semi-polar planes)^8,20,21^, and each facet has its own emission properties due to different kinetic of incorporation of In and different piezoelectric fields^8,22^. Moreover, the IQE of these non- or semi-polar QWs remained much lower than expected. Finally, the independent current injection into each separate facet to control the emission color is a severe technological challenge for applications such as RGB displays.

The NRds in this study were fabricated by a combination of nano-imprint lithography (NIL) and a mixed dry–wet etching process of GaN wafers with axial InGaN/GaN multiple quantum wells (MQWs). Contrary to bottom-up processes, the top down process enables NRds with exclusively axial InGaN/GaN MQWs from etching of InGaN/GaN MQWs layers grown under optimized condition on planar GaN. Therefore, such NRds show a single emission due to the c-plane uniform InGaN/GaN MQWs. We use a nanoimprint lithography process which is a very powerful tool because it enables the patterning of 2-in. substrates in a few minutes compared to the patterning of 1 cm^2^ in a few hours by electron or focus ion beam lithography methods used in previous studies^23–26^ which have a very high resolution (50 nm) but are expensive and time consuming and therefore not suitable for mass production. The size, position and density of the NRds are also govern by the NIL mask which allows a good homogeneity of the physical properties compared to methods based on the self-assembling of metallic nano-islands^27^ or direct deposition of silica nanoparticles^28,29^ that are fast processes but present some irregularities and dispersion in the shape and size of the NRds. Furthermore, a metal mask is preferred to obtain high aspect ratio structures by deep plasma etching. While displacement talbot lithography can pattern thick resist at the nanoscale^30^, it is still an emerging technique with a resolution limited by the wavelength illumination source, often 365 nm^30^ or 266 nm^31^, while NIL resolution can be scaled further down.

In order to change the emission wavelength, the strain relief is controlled via the diameter using a single InGaN/GaN MQWs set. Such control is achieved from 650 to 90 nm thanks to lateral wet etching in a AZ400K solution. Power dependence luminescence study is performed to show the influence of the piezoelectric field on the emission wavelength, while time-resolved (TR) luminescence is performed to enlighten any effect of the etching process on the recombination time of the charge carriers. The results are then interpreted by a phenomenological 1-D relaxation model based on an exciton potential at the center of the NRds dominated by the strain-induced piezo-electric field^32^. In the presented approach, providing the appropriate NIL mask design, the simultaneous patterning of NRds with different diameters could achieve multi-color emission from a single InGaN/GaN MQWs layer grown on GaN/c-Al_2_O_3_ in a fast and cheap single step process.

## Results and discussion

**Figure 1:**
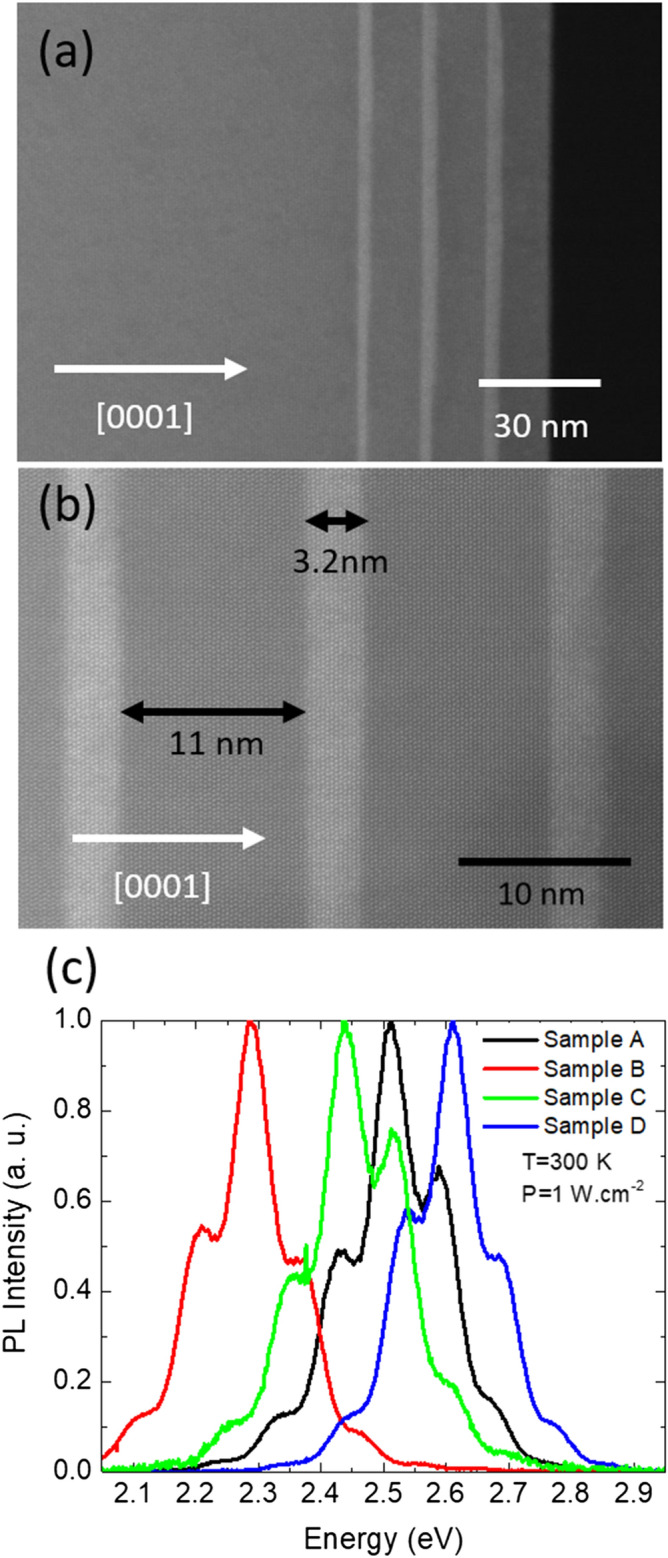
(**a**) HAADF-STEM Images of the InGaN/GaN MQWs of sample B. InGaN and GaN appear as bright and dark respectively. (**b**) High resolution image taken along the^11–20^ zone axis. (**c**) RT-PL measurement of the four InGaN/GaN MQWs layers at P = 1 W cm^−2^.

The average thicknesses and compositions of four InGaN/GaN MQWs layers were determined by ω-2θ scans and are indicated in Table 1. Their In composition varies between 17 and 21% while the thickness of the QWs vary between 1.5 and 3.5 nm. HAADF-STEM images of the InGaN/GaN MQWs of sample B prepared in longitudinal cross section by Focus Ion Beam (FIB) are shown in Fig. 1a. The three InGaN QWs have a thickness of 3.2 nm with 11 nm GaN barrier between (as seen in Fig. 1b), confirming the XRD results. The RT-PL spectra of the four MQWs layers at P = 1 W cm^−2^ are displayed in Fig. 1c, showing the different emission wavelengths.

**Table 1:** Composition, thickness of the different QWs of this study derived by XRD measurements and values of ${{E}_{0}}$, ${{B}_{m}}$ and κ for sample A, B, C and D.

Sample	A	B	C	D
QWs In composition	17	19	21	21
QWs thickness	3.5 nm	3.2 nm	2.1 nm	1.3 nm
${{E}_{0}}$ (eV)	2.8	2.78	2.82	3.0
${{B}_{m}}$ (eV)	0.32	0.42	0.34	–
κ^−1^ (nm)	27	22	19	–

**Figure 2:**
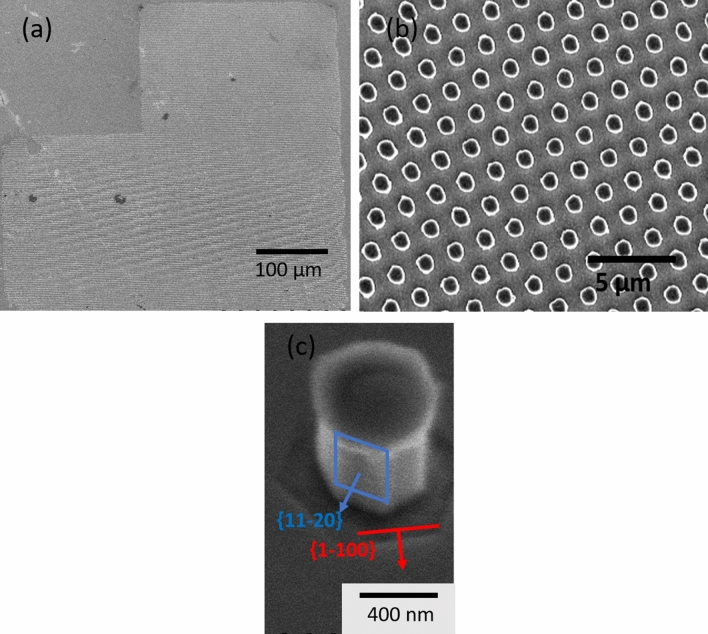
(**a**) 450 um large L-shape mesa of GaN NRds with threefold InGaN/GaN axial MQWs insertions. (**b**) Higher magnification. (**c**) SEM Image showing nanorods {1–100} facets after RIE-ICP dry etching (in red) and {11–20} facets after wet etching (in blue).

Figure 2 shows the 450 µm large L-mesa designed by NIL process (Fig. 2a), the NRds pattern (Fig. 2b) and a zoom on a typical NRd. The nanorods are along the [0001] direction and have an hexagonal shape delimited by the {11–20} planes family after dry ICP-RIE. Interestingly, due to the anisotropy of the AZ400K solution, a transition towards the {1–100} plane family is observed (Fig. 2c) during wet etching. In the following part of the study, the SEM measurement of the NRds diameter and PL measurements are always performed on the same mesa for a given sample in order to minimize errors due to In concentration or QWs thickness fluctuations on the samples.

**Figure 3:**
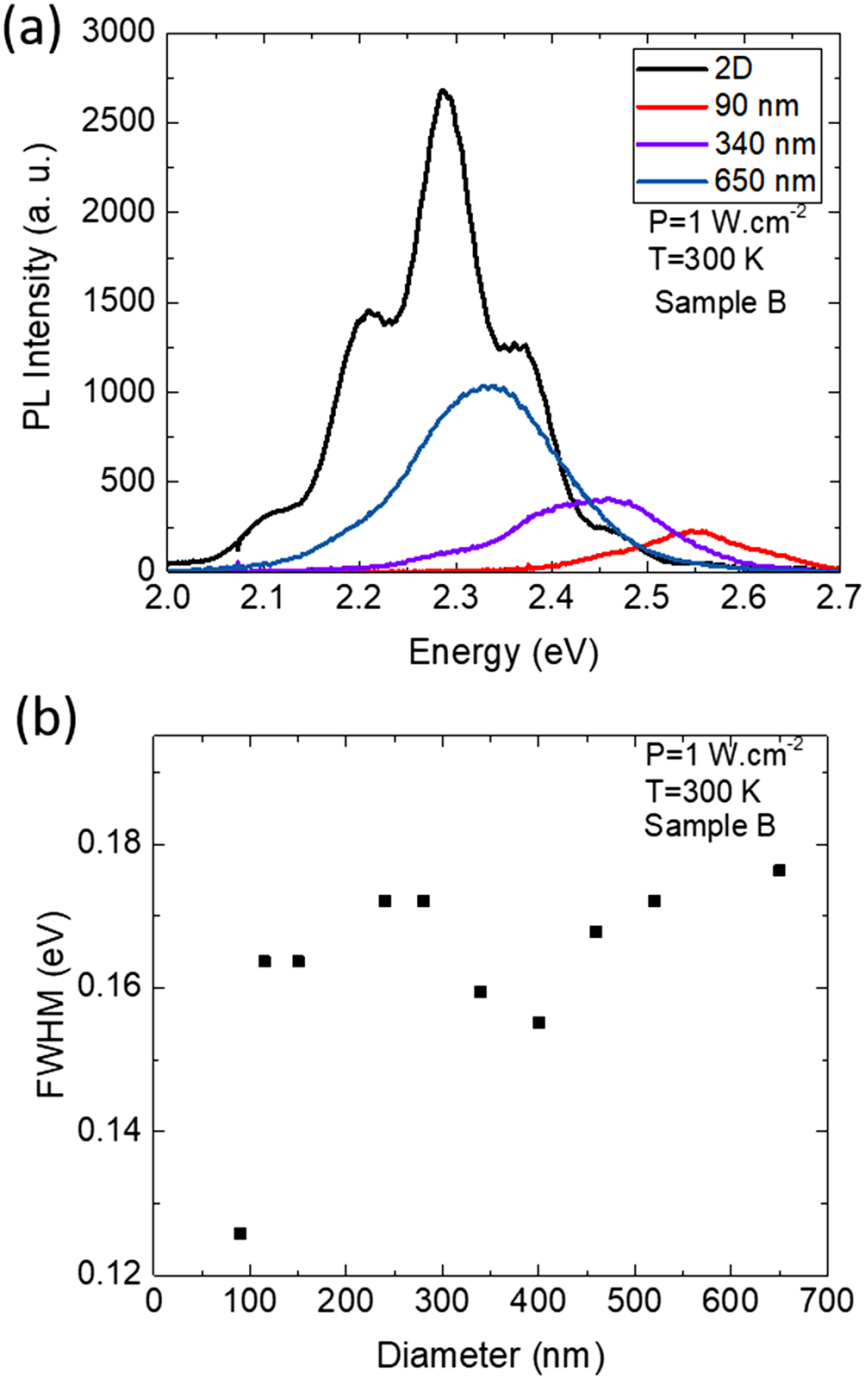
(**a**) RT-PL spectra of sample B as a function of the diameter of the NRds at P = 1 W cm^−2^. (**b**) FWHM of spectra in (**a**) as a function of the NRds diameter.

PL measurements at room temperature have been performed on InGaN NRds samples as a function of the NRds diameters after successive radial etching steps in AZ400K solution. The RT-PL spectra recorded at 1 W cm^−2^ for the sample B are presented in Fig. 3a. A 0.240 eV shift is recorded between the peak emission of the QWs layer and the 90 nm in diameter NRds. This blue shift is associated to a decrease in full width at half maximum (FWHM) as the diameter shrinks (Fig. 3b) although a two emissions component is suggested in intermediary sized NRds as illustrated by the tails at lower energy in Fig. 3a and often attributed to inhomogeneous strain in the NRds^33^.

**Figure 4:**
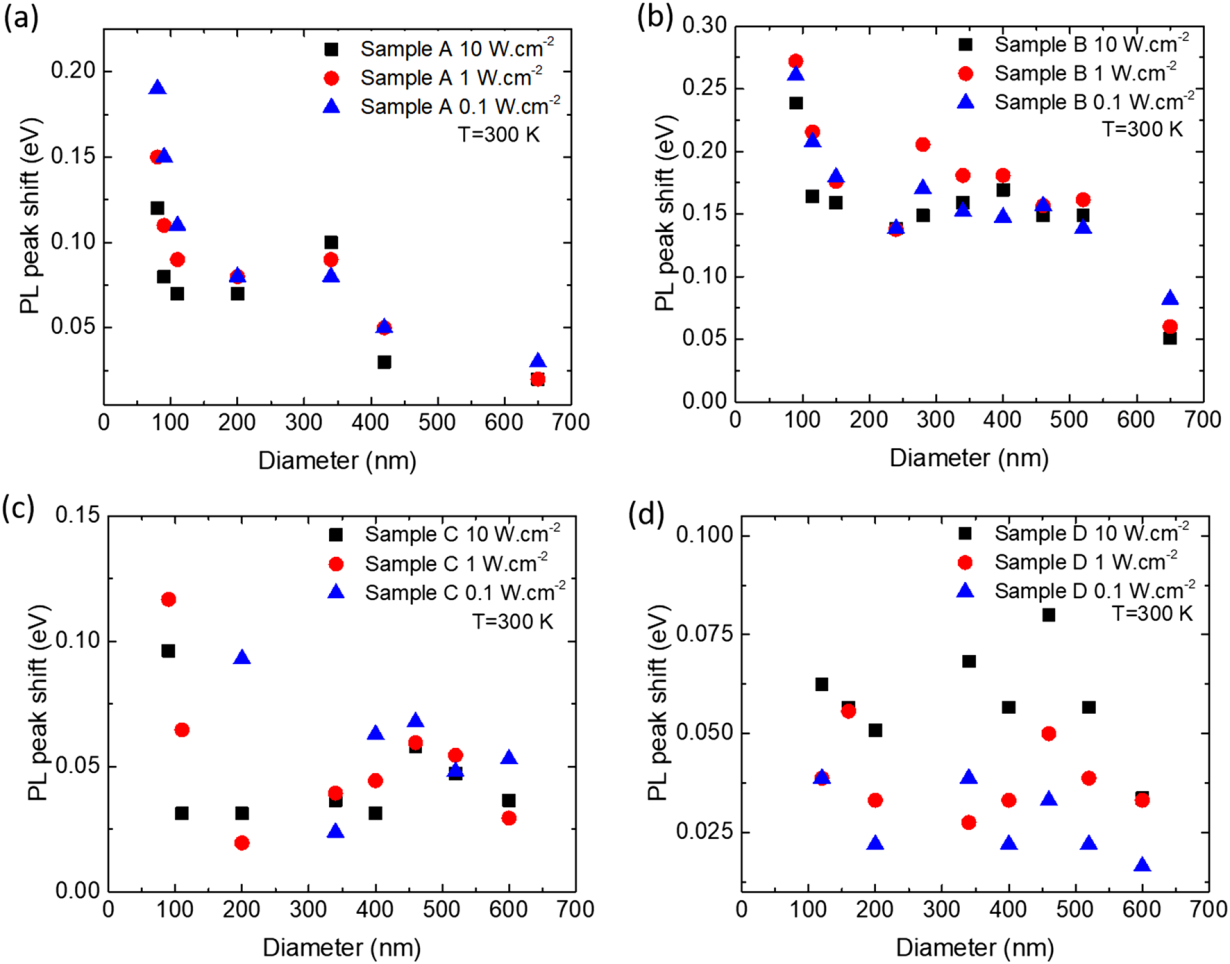
RT-PL peak shift of threefold InGaN/GaN MWQs with different indium concentration and thicknesses in GaN NRds for excitation powers of P = 0.1 W cm^−2^, 1 W cm^−2^, 10 W cm^−2^. (**a**) Sample A. (**b**) Sample B. (**c**) Sample C. (**d**) Sample D.

Power dependence PL measurements have been performed and The PL peak shifts (E_NRDs_ − E_layer_) of the emission of NRds (E_NRDs_) compared to their relative reference layers (E_Layer_) as a function of the diameter are displayed in Fig. 4b for the three following excitation powers: 10 W cm^−2^, 1 W cm^−2^ and 0.1 W cm^−2^. A first observation is that the PL emission is progressively shifted towards the blue part of the spectrum when the diameter of the NRds decreases. This shift is not linear with the diameter, but increases drastically when the diameter of the NRds reaches about 200 nm.

A systematic comparison between the samples with different In compositions and QW thicknesses has been performed. Power dependent PL measurements (in Supplementary informations) at room temperature show that for a given diameter, when the excitation power is increased from 0.1 to 10 W cm^−2^, the emission wavelength is shifted towards the blue part of the spectrum. Figure 4 also shows that at a given excitation power, the blueshift is strongly dependent on the InGaN/GaN MQWs structure. Assuming that the blueshift of the emission is due to the decreasing piezoelectric field thanks to strain relaxation in the MQWs as the diameter of the NRds shrink, one would expect that a high In composition and thick QWs induce a larger blueshift. For example, a maximum blueshift of 0.19 eV is measured for InGaN MQWs of sample A (Fig. 4.a) while the blueshift is 0.26 eV for InGaN MQWs of sample B (Fig. 4b), and a blueshift of 0.09 eV and 0.03 eV are measured for samples C (Fig. 4c) and D (Fig. 4d) at P = 1 W cm^−2^. However, in our case, due to small variations of InGaN/GaN MQWs composition and thickness more data would be required to separate and quantitively evaluate the effect of the InGaN/GaN MQWs composition and thickness on the blueshift. Obtaining homogeneous InGaN/GaN MQWs with strong emission over a wide range of thicknesses and compositions is not experimentally straightforward and it would need to deviate from the systematic study by changing more than one parameter at each growth. We explain these results by the inclined band profile in the InGaN QWs due to the piezoelectric field generated by the GaN/InGaN/GaN interfaces. At high excitation energy, the screening of the piezoelectric field by generated charged carriers leads to flattened bands and thus to a higher emission energy. In the opposite way, at a low excitation power of 0.1 W cm^−2^, the photo generated carrier density is low and the emission is strongly affect by the strain induced piezo-electric fields.

**Figure 5:**
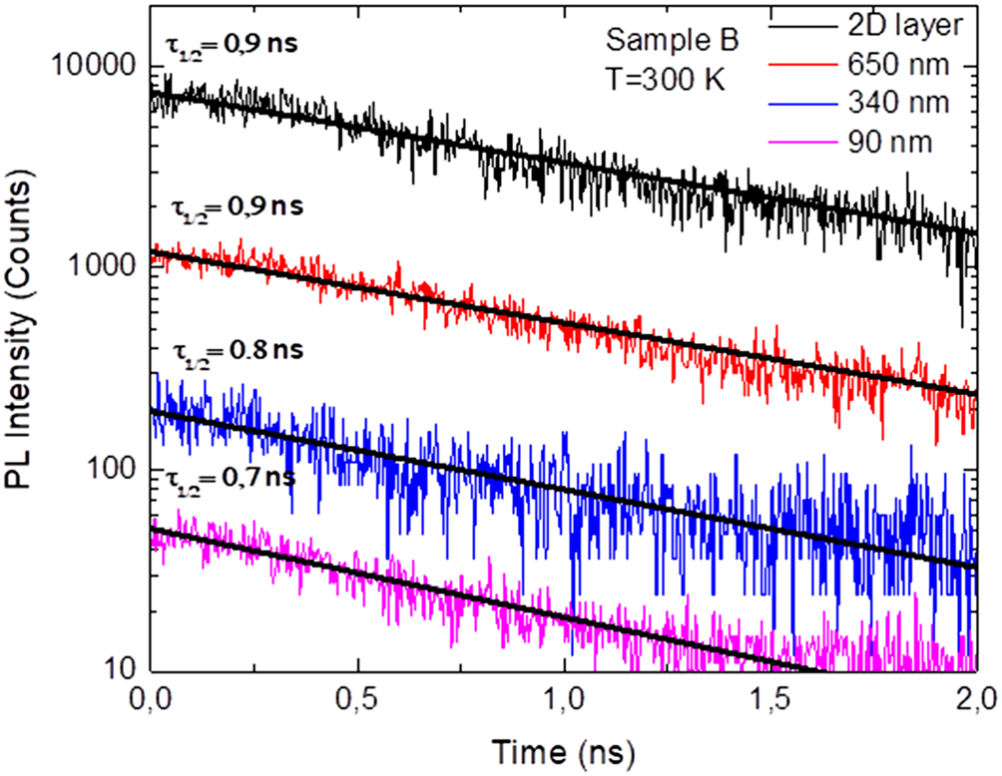
Time-resolved PL lifetime measurements at room temperature of sample B as a function of the diameter of the NRds and associated monoexponential fitting curves.

The marked decrease of strain observed by power dependence RT-PL measurements in the NRds structure should be translated in an increase of oscillator strength which is correlated to an increase of the radiative decay rate of the photoluminescence. In order to investigate qualitatively the NRds structures in terms of efficiency, TRPL measurements have been performed at room temperature. TRPL transients of the samples after successive etchings steps of NRDs ensembles have been performed, Fig. 5 shows the data from sample B. The transients are stretched exponential. A stretched exponential TRPL decay is often attributed to an inhomogeneous material, as in our case, InGaN/GaN MQWs embedded in GaN NRds. The decay time of the PL of the sample decreases when the diameter of the NRds is reduced until 90 nm.

**Figure 6:**
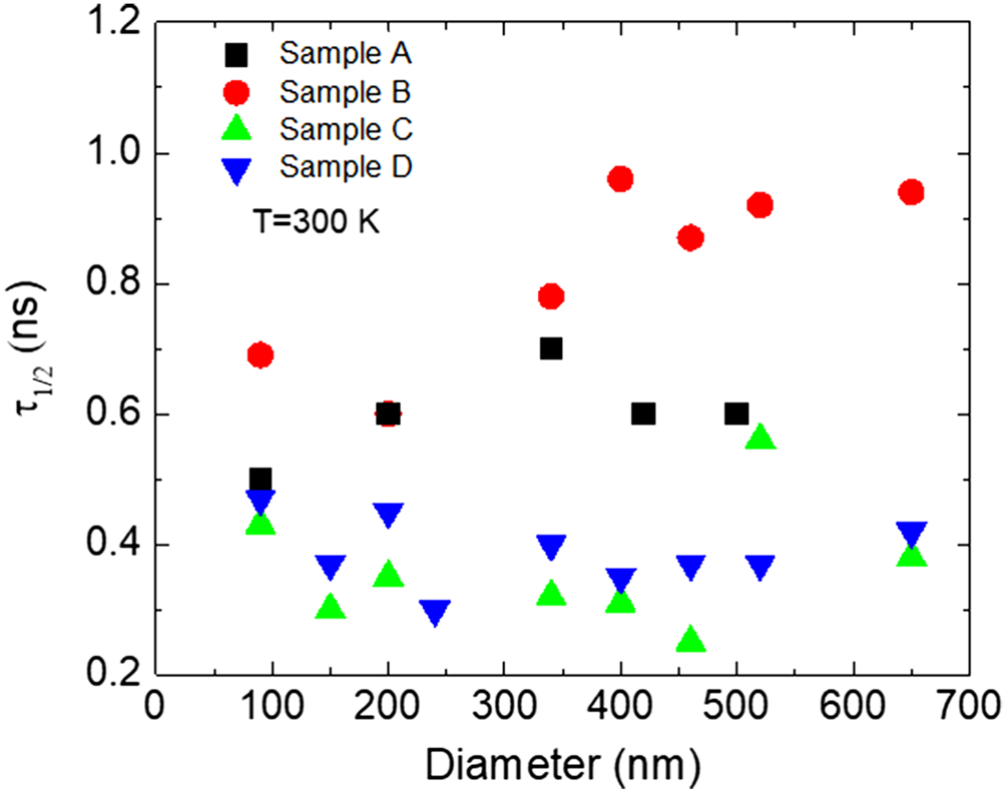
Half intensity decay time at room temperature of the four samples A, B, C and D of GaN NRds with threefolds InGaN/GaN axial MQWs with different In compositions and QWs thicknesses as a function of the diameter of the NRds.

Deconvolution of such transients can be tricky. In order to characterize the TRPL decay, we use as decay time τ_1/2_ the time needed to reach the half maximum intensity. Figure 6 show the measured half intensity TRPL decay time for all the samples for the full range of diameters. The data agree with the intentsity induced wavelength shift in Fig. 4. The decrease of the decay time with the diameter is stronger for the InGaN MQWs with the more strain, since the separation of the hole and electron wave function is the main cause for longer lifetimes. Hence a clear decrease is observed for the sample A and B, a more subtle one is observed for InGaN/GaN MQWs samples with either lower In composition or thinner QWs: Sample C and D show a rather constant decay rate with the diameter. Also, InGaN/GaN MQWs with a higher In content are expected to have a higher decay time due to larger internal fields. The TRPL spectra of the references 2D MQWs samples have also been recorded and overall, the decay time are close to the value of the post-NIL processed samples with NRds of 650 nm in diameter indicating that the NIL/dry etching process does not produce a significantly higher defect density in the NRds. A similar result can be concluded about the lack of defect generation during the successive etching steps of the NRds and reduction of strain in the InGaN/GaN QWs.

**Figure 7:**
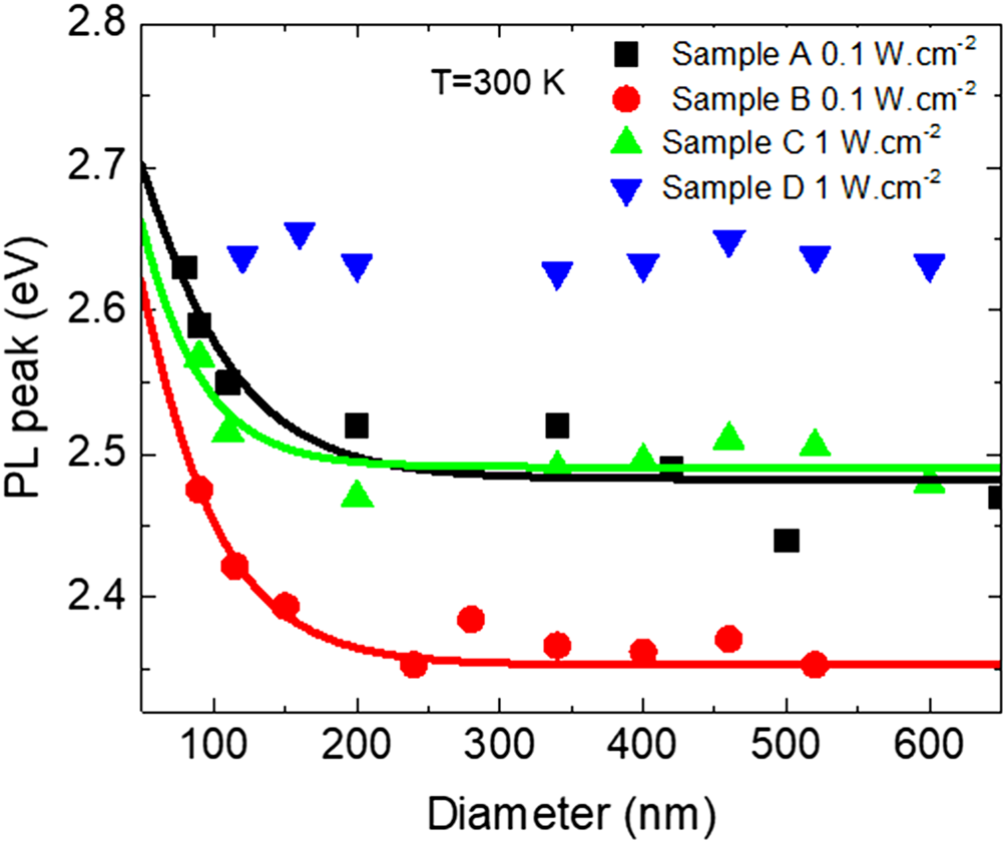
RT-PL peak position of NRds ensembles of sample A, B, C, D and associated fit (in solid line) with 1-D relaxation model represented by Eq. (1). Fit of sample D is absent because no significant strain relaxation has been observed.

**Figure 8:**
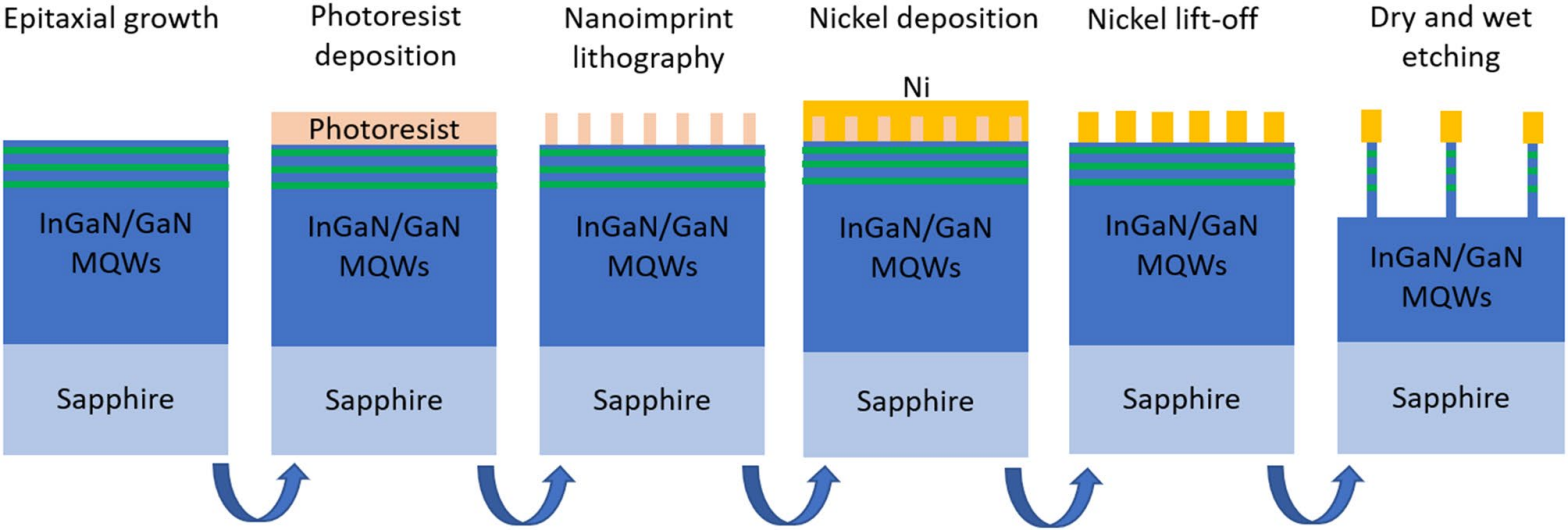
Flowchart of the nanofabrication of GaN NRds with axial InGaN/GaN MQWs by nanoimprint lithography and a mixed dry ICP wet etching process.

A phenomenological model has been developed by Zhang et al. in Ref^32^ to link the emission energy with the diameters of the NRds. It is based on a lower potential profile at the centre of the NRds induced by the piezoelectric polarization and does not take into account the spontaneous polarization. Following this model, the excitation dependant emission energy E near the centre of the nanorods of the InGaN/GaN MQWs and the diameter D of the NRds are related at low excitation density by Eq. (1) below:

1 $$E={E}_{0}-{B}_{m}\left(1-\mathit{sec}h\frac{\kappa D}{2}\right),$$

where ${E}_{0}$ is the exciton energy of a QW without fields, in the case of an infinitely thin NRd with $D\to 0$ where the strain is fully relaxed. ${B}_{m}$ is the excitation density dependant energy shift of the emission between the fully relaxed case and the fully strained case (2D layer). The constant κ^−1^ is the characteristic length of the region in the InGaN/GaN QWs sidewalls where the compressive strain can be considered as fully relaxed. The constant ${E}_{0}$ can be determined by the thickness and composition of the InGaN QWs, while ${B}_{m}$ and κ are obtained by fitting Eq. (1) with the experimental data of Fig. 7 for P = 0.1 W cm^−2^ or P = 1 W cm^−2^ when sufficient data for low diameters is missing. The fitting results are in Table 1, while Fig. 8 shows good agreement for sample A, B, and C of the solid line (fit) with the measure data. The sample D showing no change in emission; hence no fit was possible.

The characteristic relaxation lengths $$ {\raise0.7ex\hbox{$1$} \!\mathord{\left/ {\vphantom {1 \kappa }}\right.\kern-\nulldelimiterspace} \!\lower0.7ex\hbox{$\kappa $}} $$ between 19 and 27 nm are consistent with the value reported by Kawakami et al.^33^ and also found by Zhang et al.^32^ showing a non-uniform strain relaxation in the NRds. The dependence of $$ {\raise0.7ex\hbox{$1$} \!\mathord{\left/ {\vphantom {1 \kappa }}\right.\kern-\nulldelimiterspace} \!\lower0.7ex\hbox{$\kappa $}} $$ on the QWs compositions and QWs thickness and composition as also observed by Teng et al.^23^. The model describes well the experimental results and also indicates that further blue-shift is possible by further reducing the NRds diameter. This approach is very interesting since it has been demonstrated in EBL defined NRds that by reducing further the nanorods diameters until 50 nm^24,25^, green and blue emission could be achieved from amber emitting InGaN/GaN MQWs. However, in our case, the fixed pitch of 1860 nm of the NIL mask does not allow to collect enough PL signal when the diameter of the NRds decreases below 80 nm.

## Conclusion

Threefold InGaN/GaN MQWs with different QW thicknesses and compositions emitting in the blue and yellow/green part of the visible spectrum have been grown by MOVPE on c-GaN/Al_2_O_3_ substrates. Realization of NRds with axial InGaN/GaN MQWs insertions was done by a top-down process consisting in NIL and a mixed dry and wet etching process. NRds with a diameter of 650 nm could be obtained and their diameter further reduced by wet-etching. The emission wavelengths of the samples show a significant blueshift compared to the reference samples which is controlled by the NRds diameter and enable diameter-tuned yellow (2.28 eV) to blue emission (2.54 eV) (543 nm to 488 nm). Power dependence PL and time-resolved PL studies confirm that the diameter PL-dependence is driven by the relaxation of strain induced reduction of the piezoelectric field. The blueshift of the emission correlates to the reduction of the PL lifetime, hinting at better quantum efficiency when the NRds diameter is reduced. The experimental data is consistent with a 1-D based strain relaxation model where the piezoelectric field is preponderous. Furthermore, the multi-color emission from the single growth step of InGaN/GaN QWs with uniform composition and thickness and the single step patterning process of NRds with different diameters by NIL enable a cost-effective process that could be promising for solving the issue of the color mixing LED devices.

## Experimental method

### Growth of InGaN/GaN quantum wells

Growth of the InGaN/GaN quantum wells (QWs) was performed in a 3 × 2 inch closed coupled showerhead EpiQuest metal–organic vapour phase epitaxy (MOVPE) reactor on 1.8 µm thick non-intentionally doped (0001)-GaN/Al_2_O_3_ templates. N_2_ was used as a carrier gas and the temperature was varied between 720 and 740 °C during the growth of the InGaN/GaN MQWs. Trimethyl-indium (TMIn, 1.7 Pa) and triethyl-gallium (TEGa, 1.8 Pa) were used as In and Ga precursors. Following the growth of the InGaN QWs, a 1 nm thick protective GaN layer was grown at the same temperature and with the same TEGa flow rate than the underlying InGaN QW. The temperature was then increased to 830 °C in order to proceed to the growth of the 10 nm thick GaN barrier. At this point, H_2_ was added in the carrier gas with a concentration of 1% in order to avoid In incorporation in the barriers. A total of three InGaN/GaN QWs were grown. During growth, the reactor pressure was kept at 200 Torr. In this study, a total of four samples have been grown by changing the growth temperature and the growth time of the InGaN/GaN QWs. For the first sample the InGaN was grown at a temperature of 740 °C for 45 s, then three other samples were grown using a lower temperature of 720 °C for 45 s, 33 s and 23 s.

### Fabrication of nanorods by a top down process

The flowchart of the nanofabrication of GaN NRds array with a threefold InGaN/GaN MQWs insertion is summarized in Fig. 8. In order to pattern the substrate with NRds, a shadow Ni mask is deposited using the nanoimprint lithography (NIL) process. First, c-GaN/sapphire templates are dryed 30 min at 200 °C on a hot plate in order to remove water. Then a thermal nanoimprint resist (MRI 7020 R from Microresist technologies GmbH) is deposited by spin coating at 5000 rpm on the sample, the thickness of the resist is 150 nm. After a 70 s baking on a hot plate at 110 °C, the sample is loaded in a 8 inches Scivax 500 thermal nanoimprint. A 4 inches polymer stamp covered by 20 nm of Ni for anti-sticking properties is then applied with a load of 20 kN for 180 s at a temperature of 155 °C. The sample is then cooled down to 40 °C and the load is removed for demoulding. The pattern of the stamp consists in 450 µm large L-shaped mesa of hexagonal array of pillars with a diameter of 460 nm and spaced each other’s from 1860 nm. The height of the pillars is 40 nm. The residual resist in the apertures (about 110 nm) is removed by reactive ion etching (RIE) using CF_4_ plasma. The operating conditions of the RIE are 20 sccm of CF_4_ gas, a plasma power of 100 W and a pressure of 5 Pa. The RIE etching rate is about 2.4 nm s^−1^. The sample is then loaded in an electron beam evaporation system (EB). 20 nm of Ni are deposited on the sample at a rate of 0.2 Å s^−1^ under a pressure of 3.0 × 10^–4^ Pa. Metal lift-off is achieved in acetone using an ultrasonic bath. A pattern of Ni dots with a diameter of 650 nm and a height of 20 nm is then successfully obtained on the sample. The sample is introduced in an inductively coupled plasma -RIE (ICP-RIE) system in order to proceed to the anisotropic etching of the GaN/InGaN MQWs layer. 30 sccm of Cl_2_ and a bias of 15 V are used for etching at a total pressure of 2 Pa, resulting in etching rates of Ni and GaN of 0.03 nms^−1^ and 100 nms^−1^ respectively. The resulting NRds are 450 nm high. In a final step, the NRds are etched laterally by a commercially available KOH buffered AZ400K solution at 80 °C to obtain the desired diameter and to remove potential ICP-RIE damages on the sidewalls. In our conditions, the lateral etching rate is about 40 nm h^−1^. Please note that for the samples presented in this study, and contrary to what is often reported in the literature^27,34^, no significant decrease of the emission intensity of our samples after the ICP-RIE step has been recorded, possibly due to its short time. At the end of the process, the Ni mask is removed in an aqua regia solution at room temperature.

### Structural characterization

Nanorods morphologies, including diameters and heights were studied by Hitachi SU-4300 scanning electron microscope (SEM) using an acceleration voltage of 5 kV and collected with a resolution of 10 nm. Additional high-resolution imaging of the InGaN/GaN MQWs structure has been performed by scanning transmission electron microscopy (STEM) using a Hitachi HD2700 STEM system with an accelerating voltage of 200 kV and a nominal probe size of 0.1 nm after obtaining a cross section by Focus Ion Beam (FIB).

### Optical characterizations

The optical properties were analyzed by power dependence PL measurements using an excitation wavelength of 405 nm and with a laser spot of about 50 µm in diameter. A 405 nm laser laser with a 47.3 ps pulse and a repetition rate of 80 MHz was used to perform the time-resolved PL (TRPL) study, the laser beam was focus onto the sample in a 50 µm diameter spot. The micro-channel plate MCP-PMT detector has a timing uncertainty of 25 ps and the contribution of electronic to the time uncertainty is 10 ps. So overall the time response of the instrumentation is dominated by the laser pulse width. X-Ray diffraction characterization in a X’pert Philips diffractometer was used to measure the thickness of the InGaN/GaN MQWs and the composition of the InGaN alloys on unpatterend parts of the wafer. All measurements of this study were performed at room temperature (RT).

## Supplementary Information

Supplementary Information.
