# Supplementary material for: Strain-induced yellow to blue emission tailoring of axial InGaN/GaN quantum wells in GaN nanorods synthesized by nanoimprint lithography
Source: Sci Rep. 2021 Mar 24;11:6754. doi: 10.1038/s41598-021-86139-9 (PMC7990954; doi:10.1038/s41598-021-86139-9)
Supplement: Supplementary file 1 — Supplementary Information. [file 41598_2021_86139_MOESM1_ESM.pdf]

Supplementary informations for: Strain-induced yellow to blue emission tailoring of axial InGaN/GaN quantum wells in GaN nanorods synthesized by nanoimprint lithography

Geoffrey Avit*,†, Yoann Robin, Yaqiang Liao, Hu Nan, Markus Pristovsek, Hiroshi Amano.

IMaSS, Nagoya University, Furo-cho, Chikusa-ku, Nagoya 464-8601, Japan

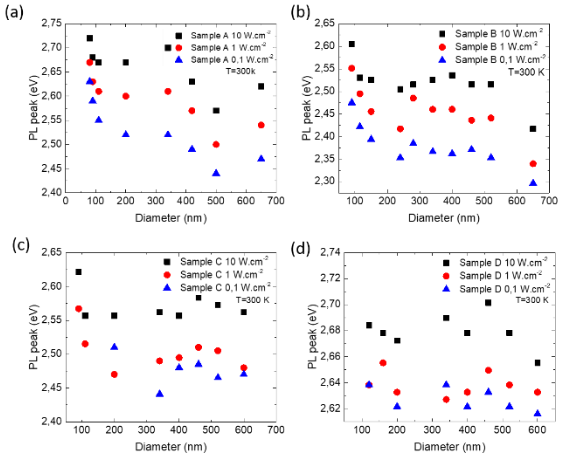

RT-PL peak of threefold InGaN/GaN MWQs with different indium concentration and thicknesses in GaN NRds for excitation powers of P = 0.1 W.cm^{-2}, 1 W. cm^{-2}, 10 W.cm^{-2}. (a) 3.5 nm thick $In_{17}Ga_{83}N$/GaN MQWs. (b) 3.2 nm thick $In_{19}Ga_{81}N$/GaN MQWs. (c) 2 nm thick $In_{21}Ga_{79}N$/GaN MQWs. (d) 1.3 nm thick $In_{21}Ga_{79}N$/GaN MQWs.

Corresponding Author

* Geoffrey Avit, IMaSS, Nagoya, University, Furo-cho, Chikusa-ku , Nagoya 464-8601, Japan
email address: geoffrey.avit@uca.fr

Present Addresses
†CNRS, UMR6602, Institut Pascal, 4 Avenue blaise Pascal, F-63178 Aubière, France.
